# Retraction Note: High-rate aluminium yolk-shell nanoparticle anode for Li-ion battery with long cycle life and ultrahigh capacity

**DOI:** 10.1038/s41467-023-41398-0

**Published:** 2023-09-13

**Authors:** Sa Li, Junjie Niu, Yu Cheng Zhao, Kang Pyo So, Chao Wang, Chang An Wang, Ju Li

**Affiliations:** 1 State Key Lab of New Ceramics and Fine Processing, School of Materials Science and Engineering, Tsinghua University, Beijing, 100084 P. R. China; 2 Department of Nuclear Science and Engineering, Massachusetts Institute of Technology, Cambridge, Massachusetts 02139 USA; 3 Department of Materials Science and Engineering, University of Wisconsin-Milwaukee, Milwaukee, Wisconsin 53211 USA; 4 Department of Materials Science and Engineering, Massachusetts Institute of Technology, Cambridge, Massachusetts 02139 USA

Retraction to: *Nature Communications* 10.1038/ncomms8872, published online 05 August 2015

The authors have retracted this article because there are a number of flaws in the figures and data. Figure 2c and the EDS mappings in Figures 2d, 2e and 2f were re-used from the authors previous work^1^ where a different material was used. Figures 4b and 4c contain sections with an unexplained blank section not matching the background or image. The EDX images in Figures 4d–4f were modified before publication. The background noise in the XRD spectra in Supplementary Figure 9 shows a much higher degree of overlap than would be expected for different cycles.

The authors sincerely apologize to the scientific community for any confusion caused by these errors. All authors agree to this retraction.
